# Egyptians' social acceptance and consenting options for posthumous organ donation; a cross sectional study

**DOI:** 10.1186/s12910-020-00490-6

**Published:** 2020-06-15

**Authors:** Ammal M. Metwally, Ghada A. Abdel-Latif, Lobna Eletreby, Ahmed Aboulghate, Amira Mohsen, Hala A. Amer, Rehan M. Saleh, Dalia M. Elmosalami, Hend I. Salama, Safaa I. Abd El Hady, Raefa R. Alam, Hanan A. Mohamed, Hanan M. Badran, Hanan E. Eltokhy, Hazem Elhariri, Thanaa Rabah, Mohamed Abdelrahman, Nihad A. Ibrahim, Nada Chami

**Affiliations:** 1 Community Medicine Research Department, Medical Research Division, National Research Centre (ID:60014618), P.O.12622, Doki, Giza, Egypt; 2 Infection Control Department, King Saud Medical City, Riyadh, Saudi Arabia; 3 Egyptian Liver Research Institute And Hospital, Mansoura, Egypt; 4 Faculty of Nursing, Mansoura University, Mansoura, Egypt

## Abstract

**Background:**

Organ donation has become one of the most effective ways to save lives and improve the quality of life for patients with end-stage organ failure. No previous studies have investigated the preferences for the different consenting options for organ donation in Egypt. This study aims to assess Egyptians’ preferences regarding consenting options for posthumous organ donation, and measure their awareness and acceptance of the Egyptian law articles regulating organ donation.

**Methods:**

A cross sectional study was conducted among 2743 participants over two years. Each participant was required to rank eleven consenting options from 1 (most preferred) to 11 (least preferred), and to report his awareness and acceptance of the seven articles of the Egyptian law of organ donation.

**Results:**

47% of the participants expressed willingness to donate their organs after death. This percentage increased to 78% when consenting options were explained to participants. “*Informed consent by donor only”* was the most preferred type of consent for one third of respondents. Awareness of the law articles regulating organ donation was relatively low ranging from 56% to 23%.

**Conclusion:**

Currently, around half of the Egyptian population agree to posthumous organ donation. This percentage could be increased significantly by raising the awareness about how the process of donation could be regulated and how the patient’s right of decision could be protected.

## Background

Organ transplantation has enabled many patients with end stage organ failure to have a longer life, a better quality of life or both. Live organ donation offers a relatively limited number of organs due to the large gap between the numbers of registered donors and the awaiting recipients [1]. In the US for instance, around 120,000 people are on the waiting list for life-saving organ transplantation [2]*.* The second source of organ donation is donors with brain stem death (posthumous organ donation). Understandably the public acceptance of this type of donation is often a matter of discourse due to the moral and legal aspects involved in its regulation and implementation [3]. The public acceptance of posthumous donation is likely influenced by the religious, cultural and social norms of the community where it is applied. Currently there is a paucity of research conducted on the public perception of posthumous organ donation in the Middle East countries [4]. In the Middle East countries, Islam is the most prevalent religion. Islam generally supports organ donation and regard it as an individual decision [5–7]. Most Muslim scholars have given permission for organ and tissue transplantation to save human lives (provided that other treatment options are considered and certain conditions are met) [8, 9]. In fact, the Quran states that "whoever saves the life of a human being, it is as if he has saved the life of all mankind" (chapter 5:32) [10]. There are, however, barriers that have hindered the legislation for posthumous organ donation in Muslim-majority countries. These barriers include the lack of consensus on equating brain death to legal death in some countries like Egypt, Morocco, Syria, Sudan, and Libya [4]. Other barriers to posthumous organ donation include the poor public awareness of the importance of organ donation and transplantation [11], the weak healthcare systems and infrastructures, the high cost and technology required and the modest government support [4]. These barriers have made living-donor organ transplantation the most widely practiced type of transplantation in the Middle East [12].

When attempting to regulate posthumous organ transplantation, it is crucial to consider the public opinion regarding the appropriate form of consenting for organ donation [13]. Due to the limited evidence published on this topic in Egypt, we conduct this study aiming to: 1) Assess the preferred form of consent for posthumous organ donation among the Egyptian population, 2) Assess the relation between the consent form preferences and the sociodemographic variables, 3) Assess the public awareness and acceptance of the law of posthumous organ donation, and 4) Assess the relation between public awareness and acceptance of posthumous organ transplantation law and their consent form preference.

## Methods

### Study Setting

Participants were chosen from two healthcare facilities in Egypt: the Egyptian Liver Hospital, Aldakhlyia governorate (*n*=1568) and the National Research Center, Giza governorate (*n*=1175).

### Study Design

A cross sectional study was conducted over a period of two years among 2743 adult participants. We included patients attending the study facilities as well as their relatives, staff members, nurses, administrative employees, workers, managers, technicians, and students.

### Sample Size and Sampling Technique

Given the lack of previous studies on our topic, we assumed the frequency of the problem that would guarantee the largest sample size to be 50 % in order to have the largest possible sample size. The Confidence limit (the absolute precision required is assumed to be 0.05 i.e. 5 percentage points out of the average). The confidence level for the interval was set at 95%. Sample size was calculated by Statcalc version 7, with a minimum of 2400 participants taking into consideration the involvement of different categories. All attendees at both study facilities were invited for inclusion in the study.

### Data Collection Types and Tools

Upon recruitment, participants were first asked whether they agree to the concept of posthumous organ donation or not. Then one-on-one counselling was performed where posthumous organ donation and the different consenting systems used for it were explained to each participant. Afterwards a questionnaire was given to participants to answer a number of questions. The questionnaire was adapted from that published by Hammami et al. [14].

The questionnaire was first explained by the research team and it was self-administrated by each participant. Illiterate respondents completed the questionnaire verbally. First, the questionnaire covered the sociodemographic characteristics. Afterwards, eleven consenting options for posthumous organ donation were given to participants who were instructed to rank them from 1 (most preferred) to 11 (least preferred). Participants were instructed to do the ranking twice, first, assuming the participant is personally involved in the donation process as a donor (hereafter referred to as the “personal preference”), and secondly assuming the participant is not involved in the donation process i.e. what is perceived to be the most appropriate for the society regardless of personal preferences (hereafter referred to as “general preference”). The eleven consenting options were: *No organ donation, Presumed consent, Informed consent by donor only (with and without medical or financial incentive), Informed consent by donor or surrogate (with and without medical or financial incentive), and Mandatory choice (with and without medical or financial incentive).*

Presumed consent was defined to participants as “Citizens must place their name on hospitals' opt-out register, otherwise their consent for donating their organs will be presumed”. The consent options were well explained by the investigators, and only participants who acknowledged understanding those options were involved in the study to minimize the bias in ranking the options.

Finally, participants were asked about their awareness and acceptance of the articles of the current Egyptian law for posthumous organ donation (currently one law with seven articles).

### Data management Analysis

Statistical Package of Social Science Software program (SPSS), version 16 was used for statistical analysis.

### Ethical Considerations

Ethical approval was obtained from the National Research Center, Egypt (registration number: 16124). Witten consent of participation was obtained from each participant. Confidentially of the collected data was maintained.

## Results

Results showed that participation of males and females was nearly equal. The age range was between 18 and 65 years old with nearly half of the sampled population in the age category of 25 to less than 45 years. Illiterates represented 8% of the study sample. Sociodemographic characteristics of participants are presented in Table 1.

**Table 1:** Characteristics of the study participants (*n*= 2743)

Characteristics	No. (%)
**Age**	
<25	695 (25)
25-<45	1184 (43)
45-65	539 (20)
>65	325 (12)
**Gender**	
Male	1282 (47)
Female	1461 (53)
**Education level**	
Postgraduate	465 (17)
University	833 (30)
Secondary	970 (35)
Primary/Preparatory	251 (10)
Illiterate	224 (8)
**Job categories**	
Professional technical managerial	869 (32)
Manual workers	742 (27)
Unemployed	601 (22)
In Education	531 (19)
**Socioeconomic characteristics**^**a**^	
Class A	299 (11)
Class B	173 (6)
Class C1	203 (7)
Class C2	759 (28)
Class D	1309 (48)
**Know organ donor**	
Yes	41 (1.5)
No	2701 (98.5)
**Know organ recipient**	
Yes	98 (3.6)
No	2642 (96.4)

^a^ Socio-economic level of the participants was calculated according to Egypt’s Central Agency for Public Mobilization And Statistics (CAPMAS) which is classified to A, B, C and D grades.

### Egyptian public perception of posthumous organ donation

**Fig. 1:**
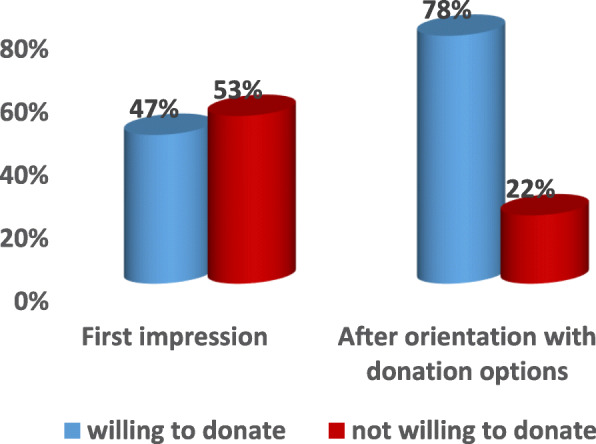
Public willingness for organ donation

The vast majority of participants (> 95%) reported not knowing an organ donor or recipient. Before the one-on-one counselling, more than half of the participants opposed posthumous organ donation (53%). However, after the counselling session this percentage dropped significantly to reach only 22% (Figure 1).

### Choice of consenting options

#### Personal preference

**Fig. 2:**
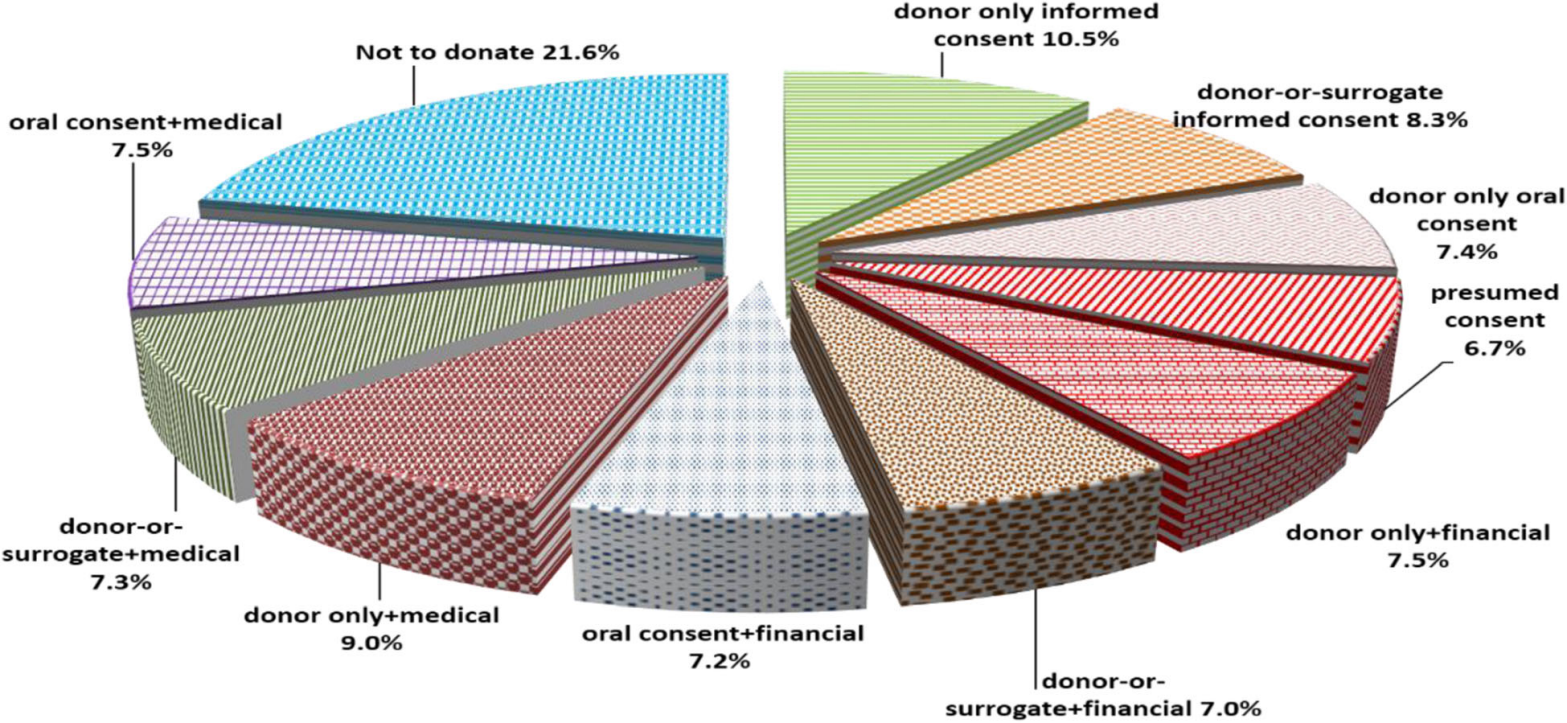
Most preferred consenting option (personal preference)

The results showed that 21.6% of the participants chose *Not to donate* as their first personal preference. Consent without incentives (*Informed consent by donor only, Informed consent by donor or surrogate, Oral consent by the donor only* and *Presumed consent*) was the first personal preference for 33% of participants. Whereas consent with medical incentive was the first choice for about 25% of participants and finally consent with financial incentive was the first choice for about 22%. *Informed consent by donor only* was ranked first by 10.5% of respondents followed by *Informed consent by donor only with a medical incentive* (9%). *Presumed consent* was found to be the least preferred consenting option with only 6.7% of respondents choosing it as their most preferred option (mode=11). (Figure 2 and Table 2).

**Table 2:** Personal preference of the eleven consenting options

	Informed consent by donor only (2743)	Informed consent by donor or surrogate (2743)	Oral consent by the donor only (2743)	Implementation of the presumed consent (2743)	Informed consent by donor only with the financial incentive (2743)	Informed consent by donor or surrogate with financial incentive (2743)	Oral consent with the financial incentive (2743)	Informed consent by donor only with the medical incentive (2743)	Informed consent by donor or surrogate with medical incentive (2743)	Oral consent by donor only with the medical incentive (2743)	Not to donate their organs after death (2743)
**1**^**st**^**Rank**	288 (10.5)	228 (8.3)	203 (7.4)	184 (6.7)	207 (7.5)	191 (7.0)	197 (7.2)	246 (9.0)	200 (7.3)	206 (7.5)	593 (21.6)
**2**^**nd**^**Rank**	348 (12.7)	254 (9.3)	251 (9.2)	214 (7.8)	251 (9.2)	233 (8.5)	208 (7.6)	308 (11.2)	238 (8.7)	231 (8.4)	206 (7.5)
**3**^**rd**^**Rank**	212 (7.7)	244 (8.9)	239 (8.7)	209 (7.6)	263 (9.6)	239 (8.7)	237 (8.6)	242 (8.8)	311 (11.3)	329 (12.0)	217 (7.9)
**4**^**th**^**Rank**	245 (8.9)	216 (7.9)	258 (9.4)	214 (7.8)	245 (8.9)	259 (9.4)	270 (9.8)	254 (9.3)	293 (10.7)	276 (10.1)	212 (7.7)
**5**^**th**^**Rank**	285 (10.4)	249 (9.1)	246 (9.0)	224 (8.2)	306 (11.2)	224 (8.2)	233 (8.5)	274 (10.0)	264 (9.6)	268 (9.8)	169 (6.2)
**6**^**th**^**Rank**	218 (8.0)	278 (10.1)	259 (9.4)	201 (7.3)	282 (10.3)	276 (10.1)	263 (9.6)	267 (9.7)	253 (9.2)	260 (9.5)	185 (6.7)
**7**^**th**^**Rank**	222 (8.1)	247 (9.0)	233 (8.5)	212 (7.7)	256 (9.3)	281 (10.2)	292 (10.6)	243 (8.9)	278 (10.1)	277 (10.1)	201 (7.3)
**8**^**th**^**Rank**	237 (8.6)	262 (9.6)	255 (9.3)	229 (8.4)	274 (10.0)	241 (8.8)	279 (10.2)	228 (8.3)	264 (9.6)	267 (9.7)	206 (7.5)
**9**^**th**^**Rank**	224 (8.2)	321 (11.7)	270 (9.8)	223 (8.1)	236 (8.6)	294 (10.7)	303 (11.1)	232 (8.5)	226 (8.2)	227 (8.3)	186 (6.8)
**10**^**th**^**Rank**	233 (8.5)	243 (8.9)	314 (11.5)	276 (10.1)	224 (8.2)	292 (10.6)	247 (9.0)	238 (8.7)	223 (8.1)	201 (7.3)	251 (9.2)
**11**^**th**^**Rank**	230 (8.4)	200 (7.3)	214 (7.8)	556 (20.3)	198 (7.2)	212 (7.7)	213 (7.8)	210 (7.7)	192 (7.0)	200 (7.3)	317 (11.6)
**Median** **25%-75%**	5 3-9	6 3-9	6 3-9	7 4-10	6 3-8	6 4-9	6 4-9	6 3-8	6 3-8	6 3-8	5 2-9
**Mean (SD)**	5.7 (3.2)	6.05 (3.09)	6.1 (3.1)	6.7 (3.3)	5.9 (3.01)	6.1 (3.06)	6.1 (3.02)	5.7 (3.1)	5.8 (3)	5.8 (3)	5.5 (3.5)
**Mode**	2	9	10	11	5	9	9	2	3	3	1

#### General preference

Regarding the general preference, *Not to donate* option was ranked first by 19.5% of respondents (mode=1). Consent with medical incentive was ranked first by 24.3% of participants whereas consent with financial incentive was ranked first by 22%. *Informed consent by donor only* was also a preferable choice with 12% of respondents ranking it first. *Presumed consent,* however, was again the least preferable option with a mode of 11 Table 3.

**Table 3:** General preference of the eleven consenting options

	Informed consent by donor only (2743)	Informed consent by donor or surrogate (2743)	Oral consent by the donor only (2743)	Implementation of the presumed consent (2743)	Informed consent by donor only with the financial incentive (2743)	Informed consent by donor or surrogate with financial incentive (2743)	Oral consent with the financial incentive (2743)	Informed consent by donor only with the medical incentive (2743)	Informed consent by donor or surrogate with medical incentive (2743)	Oral consent by donor only with the medical incentive (2743)	Not to donate their organs after death (2743)
**1st Rank**	333 (12.1)	206 (7.5)	186 (6.8)	212 (7.7)	217 (7.9)	204 (7.4)	183 (6.7)	244 (8.9)	213 (7.8)	209 (7.6)	536 (19.5)
**2nd Rank**	314 (11.4)	240 (8.7)	268 (9.8)	217 (7.9)	247 (9.0)	220 (8.0)	202 (7.4)	331 (12.1)	242 (8.8)	245 (8.9)	217 (7.6)
**3rd Rank**	221 (8.1)	280 (10.2)	244 (8.9)	203 (7.4)	263 (9.6)	259 (9.4)	213 (7.8)	290 (10.6)	293 (10.7)	289 (10.5)	188 (6.9)
**4th Rank**	223 (8.1)	254 (9.3)	255 (9.3)	188 (6.9)	225 (8.2)	235 (8.6)	272 (9.9)	285 (10.4)	311 (11.3)	294 (10.7)	201 (7.3)
**5th Rank**	277 (10.1)	218 (7.9)	248 (9.0)	217 (7.9)	312 (11.4)	210 (7.7)	266 (9.7)	258 (9.4)	247 (9.0)	273 (10.0)	217 (7.9)
**6th Rank**	233 (8.5)	233 (8.5)	253 (9.2)	219 (8.0)	264 (9.6)	304 (11.1)	282 (10.3)	254 (9.3)	251 (9.2)	275 (10.0)	175 (6.4)
**7th Rank**	226 (8.2)	249 (9.1)	225 (8.2)	247 (9.0)	255 (9.3)	301 (11.0)	305 (11.1)	214 (7.8)	273 (10.0)	248 (9.0)	200 (7.3)
**8th Rank**	240 (8.7)	272 (9.9)	221 (8.1)	222 (8.1)	267 (9.7)	244 (8.9)	281 (10.2)	232 (8.5)	256 (9.3)	260 (9.5)	248 (9.0)
**9th Rank**	209 (7.6)	333 (12.1)	271 (9.9)	205 (7.5)	256 (9.3)	205 (11.1)	276 (10.1)	226 (8.2)	242 (8.8)	239 (8.7)	181 (6.6)
**10th Rank**	223 (8.1)	253 (9.2)	340 (12.4)	263 (9.6)	237 (8.6)	358 (9.4)	225 (8.2)	218 (7.9)	245 (8.9)	209 (7.6)	272 (9.9)
**11th Rank**	244 (8.9)	205 (7.5)	232 (8.5)	550 (20.1)	200 (7.3)	203 (7.4)	238 (8.7)	191 (7.0)	170 (6.2)	202 (7.4)	308 (11.2)
**Median** **25%-75%**	6 3-8	6 3-9	6 3-9	7 4-10	6 3-9	6 4-9	6 4-9	5 3-8	6 3-8	6 3-8	6 2-9
**Mean (SD)**	5.6 (3.2)	6.1 (3.09)	6.1 (3.1)	6.7 (3.3)	5.9 (3.04)	6.1 (3.04)	6.2 (2.9)	5.6 (3.1)	5.8 (3.01)	5.8 (3.02)	5.6 (3.5)
**Mode**	1	9	10	11	5	9	7	2	4	4	1

Results were found to be more or less similar for both sexes and for both preferences (personal vs. general). Refusal of organ donation was the first choice for both males and females, both personally and generally (mode=1). *Presumed consent* was the least preferred choice followed by *Oral consent of donor* (modes=11 and 10 respectively). Difference in choice was observed when incentives were added, where females preferred consent with medical incentive as a personal preference more than males who on the other hand favored consent with financial incentive (Table 4).

**Table 4:** Personal and general preferences of the eleven consenting options according to gender

	Informed consent by donor only (2743)	Informed consent by donor or surrogate (2743)	Oral consent by the donor only (2743)	Implementation of the presumed consent (2743)	Informed consent by donor only with the financial incentive (2743)	Informed consent by donor-or surrogate with financial incentive (2743)	Oral consent with the financial incentive (2743)	Informed consent by donor only with the medical incentive (2743)	Informed consent by donor-or surrogate with medical incentive (2743)	Oral consent by donor only with the medical incentive (2743)	Not to donate their organs after death (2743)
Male: Personal preferences											
(median)	6	6	6	7	6	6	6	6	6	6	6
25%-75%	3-9	3-9	3-9	4-10	3-8	3-9	4-9	3-9	3-8	3-8	2-9
Mode	2	9	10	11	5	9	7	5	3^a^	3	1
Male: Public Norms											
(median)	6	6	6	7	6	6	6	6	6	6	6
25%-75%	3-8	3-9	3-9	4-10	3-8	3-9	4-9	3-8	3-8	3-9	2-9
Mode	2	8	10	11	5	6	5	2	4	3	1
Female: Personal preferences											
(median)	5	6	6	7	6	6	6	6	6	6	5
25%-75%	3-8	3-9	3-9	4-10	4-8	4-9	4-9	3-8	3-8	3-8	2-9
Mode	2	9	10	11	8	6	9	2	3	3	1
Female: Public Norms											
(median)	5	6	6	7	6	7	6	5	6	6	5
25%-75%	3-8	3-9	3-9	4-10	3-9	4-9	4-9	3-8	3-8	3-8	2-9
Mode	1	9	10	11	5	9	7	2	4	4	1

^a^ Multiple modes exist. The smallest value is shown

When the results were analyzed across the education level groups, *Refusal of organ donation* was the first choice of all levels of education (mode=1) except for participants with post-graduate degrees who chose *Informed consent by the donor* as their first choice, both personally and generally (mode=1). *Presumed consent* was the least preferred choice for all education groups (mode =11) except for the uneducated group general preference (Table 5).

**Table 5:** Personal and general preferences of the eleven consenting options according to education

	Informed consent by donor only (2743)	Informed consent by donor or surrogate (2743)	Oral consent by the donor only (2743)	Implementation of the presumed consent (2743)	Informed consent by donor only with the financial incentive (2743)	Informed consent by donor-or surrogate with financial incentive (2743)	Oral consent with the financial incentive (2743)	Informed consent by donor only with the medical incentive (2743)	Informed consent by donor or surrogate with medical incentive (2743)	Oral consent by donor only with the medical incentive (2743)	Not to donate their organs after death (2743)
Uneducated: Personal preferences											
median	6	6	6	6	6	6	6	6	5	6	5
Mode	2	5	8	11	7	7	1	8	2	3	1
25%-75%	3-9	4-8	3-9	4-9	4-9	3-9	3-9	3-9	3-8	3-9	3-9
Uneducated: Public Norms											
median	6	6	6	6.5	6	7	6	6	6	5	5
Mode	6	10	11	2	10	9	5	4	5^a^	5	1
25%-75%	3-8	3-9	3-9	3-9	3.25-9	3-9	4-8	4-9	3-8	3-8	2-8
Primary and preparatory education: Personal preferences											
median	6	6	7	7	5	5	6	7	6	6	5
Mode	6	10	8	11	3	2^a^	2	9	5	7	1
25%-75%	4-9	3-9	4-9	3-9	3-8	3-9	3-9	4-9	4-9	3-8	2-8
Primary and preparatory education: Public Norms											
median	6	6	6	6	5	5	6	6	6	7	6
Mode	10	5	6^a^	11	2	4	4	1	1^a^	7	1
25%-75%	4-9	3-9	3-9	3-10	3-8	3-8	4-9	3-9	3-9	4-9	3-9
Secondary education: Personal preferences											
median	6	6	6	7	6	6	6	6	6	6	5.5
Mode	2	9	10	11	2	4	4	6	3	6	1
25%-75%	3-9	3-9	3-9	3-10	3-8	3-9	3-9	3-9	3-8	3-8	2-9
Secondary education: Public norms											
median	6	6	6	7	6	6	6	6	6	6	5
Mode	1	9	10	11	5	6	4	2	7	6	1
25%-75%	3-9	3-9	3-9	4-9	3-9	3-9	3-9	3-8	4-9	3-9	2-8
University: Personal preferences											
median	5	6	6	8	6	7	7	5	5	5	4
Mode	2	9	10	11	6	6	8	2	3	3	1
25%-75%	2-8	3-9	4-9	5-11	4-8	4-9	4-9	2-8	3-9	3-8	1-9
University: Public norms											
median	5	6	7	8	6	6	7	5	5	5	5
Mode	2	9	10	11	5	6	7	2	4	4	1
25%-75%	2-8	3-9	4-9	4-11	4-8	4-9	4-9	3-8	3-8	3-8	1-9
Postgraduate: Personal preferences											
median	5	6	5	7	6	7	6	5	6	6	6
Mode	1	2	4	11	3	9	7	5	8	3	11
25%-75%	2-9	3-8	3-8	4-10	3-9	4-9	4-9	3-8	3-8	3-8	3-10
Postgraduate: Public norms											
median	5	6	6	7	6	6	7	5	5	5	7
Mode	1	2^a^	5	11	5	9	7	2	4	4	11
25%-75%	2-8	3-9	3-9	4-10	4-8	4-9	4-9	3-8	3-8	3-8	3-10

^a^ Multiple modes exist. The smallest value is shown

When the results were analyzed across the employment level groups, results were more or less similar across groups. *Refusal of organ donation* was the first choice for employed participants (professional and manual jobs) and students (mode=1), while it was the least preferred choice for unemployed participants (mode=11). *Presumed consent* was the least preferred choice for most participants (mode=11). Difference in choice was observed when incentives were added, where participants with manual jobs preferred consent with financial incentive as a personal preference more than participants with professional jobs who on the other hand favored consent with medical incentive (Table 6).

**Table 6:** Personal and general preferences of the eleven consenting options according to employment

	Informed consent by donor only (2743)	Informed consent by donor or surrogate (2743)	Oral consent by the donor only (2743)	Implementation of the presumed consent (2743)	Informed consent by donor only with the financial incentive (2743)	Informed consent by donor or surrogate with financial incentive (2743)	Oral consent with the financial incentive (2743)	Informed consent by donor only with the medical incentive (2743)	Informed consent by donor or surrogate with medical incentive (2743)	Oral consent by donor only with the medical incentive (2743)	Not to donate their organs after death (2743)
Employed in professional, technical, managerial position: Personal preferences											
median	5	6	6	9	6	7	7	5	6	5	4
Mode	2	9	10	11	6	7	7	2	3	3	1
25%-75%	2-8	3-9	4-9	5-11	3-8	4-9	4-9	3-7	3-8	3-8	1-9
Employed in professional, technical, managerial position: Public Norms											
median	5	7	7	9	6	6	7	5	5	5	5
Mode	1^a^	9	10	11	5	6	7	2	3	4	1
25%-75%	2-8	3-9	4-9	5-11	3.5-8	4-9	4-9	2-8	3-8	3-8	1-10
Working in jobs categorized as skilled or non skilled manual workers: Personal preferences											
median	6	6	6	6	6	6	6	6	6	6	6
Mode	2	5	6	11	2^a^	3	4	10	8	6	1
25%-75%	3-9	3-9	4-9	3-9	3-8	3-9	4-9	3-9	3-8	3-8	3-9
Working in jobs categorized as skilled or non skilled manual workers: Public Norms											
median	6	6	6	6	6	6	6	6	6	6	5
Mode	11	4^a^	10	11	2	3	4	3	5	6^a^	1
25%-75%	3-9	3-9	3-9	3-9	3-9	3-9	3-9	3-9	3.75-9	3-9	3-9
Student: Personal preferences											
median	6	6	6	7	6	6	6	6	6	6	5
Mode	2	9	6	11	5^a^	10	9	9	5	3	1
25%-75%	3-9	3-9	3-9	4-10	3-9	3-9	3-9	3-9	3-9	3-8	2-8
Student: Public norms											
median	5	6	6	7	6	7	6	6	6	6	5
Mode	1	9	2	11	9	8	9	4	4	6	1
25%-75%	3-9	3-9	3-9	4-10	3-9	4-9	4-9	3-8	3-9	3-8	2-8
Unemployed: Personal preferences											
median	6	6	6	7	6	6	6	6	6	6	6
Mode	5	1	8	8	5^a^	10	2	7^a^	3	7	11
25%-75%	3-9	3-8	3-9	4-9	4-8	4-9	3-9	3-9	3-8	3-9	3-9
Unemployed: Public norms											
median	6	6	6	6	6	6	6	6	6	6	6
Mode	1	6^a^	10	7	3^a^	9	2	3	4	4	11
25%-75%	3-9	3-9	4-9	3-9	3-9	3-9	4-9	3-9	3-9	3-9	3-9

^a^ Multiple modes exist. The smallest value is shown

*Refusal of organ donation* was the first choice for all socioeconomic levels (mode=1) except for the most deprived group (D). On the other hand, the least preferred choice for all socioeconomic levels was the *Presumed consent* (mode=11) except for the most deprived group (D) (Table 7).

**Table 7:** Personal and general preferences of the eleven consenting options according to socioeconomic level

	Informed consent by donor only (2743)	Informed consent by donor or surrogate (2743)	Oral consent by the donor only (2743)	Implementation of the presumed consent (2743)	Informed consent by donor only with the financial incentive (2743)	Informed consent by donor or surrogate with financial incentive (2743)	Oral consent with the financial incentive (2743)	Informed consent by donor only with the medical incentive (2743)	Informed consent by donor-or surrogate with medical incentive (2743)	Oral consent by donor only with the medical incentive (2743)	Not to donate their organs after death (2743)
A: Personal preferences											
median	4	6	6	10	6	7	8	4	5	5	5
Mode	1	9	10	11	5	9	9	2	4	3	1
25%-75%	2-7	3-8	4-9	7-11	5-8	6-9	6-9	2-6	3-7	3-7	1-11
A: Public Norms											
median	4	7	6	10	6	7	7	4	5	5	6
Mode	1	9	9	11	5	6	7	2	4	3	1
25%-75%	2-8	4-9	3-9	7-11	5-8	6-9	6-9	2-6	3-7	3-7	1-11
B: Personal preferences											
median	5	7	7	8	6	7	7	5	5	5	5
Mode	2	9	10	11	6	7	7	2	3	3	1
25%-75%	2-8	3-9	4-9	4-11	4-9	5-8	5-9	2-8	3-7	3-8	1-9
B: Public Norms											
median	5	7	8	8	6	6	7	4	5	4	6
Mode	1	9	10	11	5	6	7^a^	2	3	4	1
25%-75%	2-8	3-9	4-10	5-11	4.5-9	4-8	5-9	2-7	3-8	3-7	1-9
C1: Personal preferences											
median	5	8	8	11	6	7	7	5	5.5	5.5	1
Mode	2	9	10	11	8	7	8	2	7	4	1
25%-75%	2-8	5-9	4-10	9-11	3-8	4-9	4-9	3-6	4-7	4-8	1-4
C1: Public norms											
median	5	8	7	11	6	6	6	5	6	6	1
Mode	2	9	10	11	2	3^a^	4	2	7	4^a^	1
25%-75%	2-8	4-9	4-10	8-11	3-8	4-9	4-8	3-6	3-7	4-8	1-7
C2: Personal preferences											
median	6	6	6	6	6	6	6	6	6	6	6
Mode	8^a^	6	6	11	5	10	11	9	10	3	1
25%-75%	3-9	3-9	3-9	3-9	3-8	3-9	3-9	4-9	3-9	3-9	3-9
C2: Public norms											
median	6	6	6	6	6	6	6	6	6	6	5
Mode	7	8	2^a^	11	3	7^a^	4	6	10	9	1
25%-75%	3-9	3-9	3-9	3-9	3-9	3-9	4-9	3-9	3-9	3-9	2-9
D: Personal preferences											
median	6	6	6	6	6	6	6	6	6	6	6
Mode	2	6	8	5^a^	4	4	7	1	8	7	3
25%-75%	3-9	3-9	3-9	3-9	3-9	3-9	3-9	3-9	3-9	3-9	3-9
D: Public norms											
median	6	6	6	6	6	6	6	6	3	6	6
Mode	11	3	5	11	10	9	7	10	8	6	8
25%-75%	3-9	3-9	3-9	3-9	3-9	3-9	3-9	3-9	3-9	3-9	3-9

^a^ Multiple modes exist. The smallest value is shown

### Awareness and acceptance of the posthumous organ donation law

The percentage of participants aware of the articles of the posthumous organ donation law was relatively low, ranging from 56% to 23%. Article number 3 (*The expenses conducting the organ transplantation will be covered by the government for those who cannot afford it in accordance with the regulations issued by the Minster of Health*) received the highest awareness rate whereas Article number 1 (*Possibility of organ donation to any child of an Egyptian mother and a foreign father*) showed the lowest awareness rate.

**Fig. 3:**
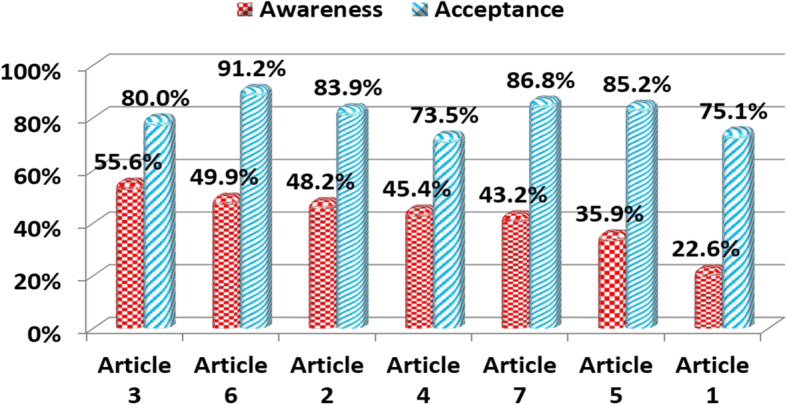
The Awareness and the acceptance of the laws of posthumous organ donation. Key points addressed under each article: 1 - Possibility of organ donation to any child of Egyptian mother and a foreign father. 2 - Severe penalties for those involved in illegal for-profit transplantation (including long jail sentences and considerable fines). 3 - The expenses of organ transplantation for those who cannot afford are to be covered by the government. 4 - Transfer of an organ or tissue from a dead body is not to be allowed until death is confirmed by a triple committee of specialized doctors in neurosurgery, cardiothoracic surgery and anesthesia. 5 - Live organ donation is only allowed between Egyptians and without any payment to the donor. 6 - Organ donation could be permitted to non-relatives if the patient is in urgent need for transplantation. 7 - Procurement of an organ or part of an organ or tissue from a person without valid evidence of death would be punished by the penalty of premediated murder as stipulated in article 230

Acceptance rates of the law articles were high ranging from 91% to 73.5%. The highest acceptance rate was found to be for Article number 6 (*Organ donation could be permitted to non-relatives if the patient is in urgent need for the transplantation*) and the lowest was for Article number 4 (*The law does not allow the transfer of an organ or tissue from a dead body until death is confirmed by a triple committee of specialized doctors in neurosurgery, cardiothoracic surgery and anesthesia*) (Figure 3).

About half of the participants who refused to donate their organs after death were unaware of the laws that regulate organ donation, while most of the participants who choose to donate their organs after death were aware of three or more law articles (p<0.001). Regarding the acceptance of those laws, almost all participants accepted them and this relation was statistically significant (*p*<0.001) (Table 8).

**Table 8:** Relation between awareness and acceptance of law articles among those who chose the different consenting options as their first choice

	Types of consent (2743)										
	Informed consent by donor only	Informed consent by donor or surrogate	Oral consent by the donor only	Implementation of the presumed consent	Informed consent by donor only with the financial incentive	Informed consent by donor or surrogate with financial incentive	Oral consent with the financial incentive	Informed consent by donor only with the medical incentive	Informed consent by donor or surrogate with medical incentive	Oral consent by donor-only with the medical incentive	Not to donate their organs after death
**Awareness**											
Not aware	71 (21.5)	(12.7)	27 (14.6)	22 (10.4)	19 (8.8)	17 (8.4)	13 (7.1)	42 (17.3)	23 (10.9)	29 (14.1)	257 (48.0)
Aware <3	66 (19.9)	30 (14.6)	23 (12.4)	31 (14.6)	38 (17.5)	23 (11.3)	21 (11.5)	32(13.2)	32 (15.2)	25 (12.1)	89 (16.6)
Aware 3 or more	194 (58.6)	149 (72.7)	135 (73.0)	159 (75.0)	160 (73.7)	163 (80.3)	149 (81.4)	169 (69.5)	156 (73.9)	152 (73.8)	189(35.3)
Total	331 (100)	205 (100)	185 (100)	212 (100)	217 (100)	203 (100)	183 (100)	243 (100)	211 (100)	206 (100)	535 (100)
***P*<0.001**											
**Acceptance**											
Do not accept	1 (0.3)	2 (1.0)	1 (0.5)	1 (0.5)	0 (0.0)	2 (1.0)	0 (0.0)	1 (0.4)	1 (0.5)	4 (1.9)	17 (3.2)
Accept <3	6 (1.8)	8 (3.9)	1 (0.5)	3 (1.4)	3 (1.4)	2 (1.0)	1 (0.5)	4 (1.6)	2 (0.9)	4 (1.9)	15 (2.8)
Accept 3 or more	326 (97.9)	195 (95.1)	182 (99.0)	207 (98.1)	214 (98.6)	200 (98.0)	182 (99.5)	238 (97.9)	210 (98.6)	199 (96.2)	501 (94.0)
***P*<0.001**											

## Discussion:

To our knowledge, this is the first study to assess Egyptians’ preferences on consenting options for posthumous organ donation as well as the awareness and acceptance of the Egyptian law regulating organ donation.

The initial results showed that only half of the participants agreed to posthumous organ donation (47%). However, after explaining the process of donation, its regulation and consenting form to them, this percentage increased significantly to reach 78%. These results should be viewed in light of other studies addressing the same topic in other countries. Refusal of organ donation was found to be around 13% in Saudi Arabia [15] 42% in the UK (the highest in Europe) [16] and 38% in Pakistan [17]. It is important to note though that a respondent refusal rate likely differs from the actual refusal rate when individuals are placed in a situation to actually make a decision to donate. In the UK, surveys demonstrate a refusal rate of less than 10% but that increases significantly when individuals are in the position of donating in real life [16]. The problem of converting an intention to donate in the abstract into a formal behavior change could be a significant barrier.

Our results suggest that increasing awareness of posthumous organ donation could significantly increase its acceptance among the public. These results are supported by other interventional studies for behavioral changes conducted in Egypt which showed substantial success of behavioral change interventions among communities especially the rural and closed ones [18–22]. This is further supported by our findings that awareness of organ donation laws was associated with more willingness to donate. It was found that the majority of those accepting posthumous donation were aware of three or more of the current donation law articles. Similarly, Mossialos et al. found that individuals' awareness of the legislation had a significant effect on their willingness to donate [23]. This suggests that efforts to improve educational programs and informational campaigns on the social and health benefits of organ donation could contribute to increasing the number of donations. It is worth noting though that these results were not replicable in other studies [24].

The percentages of individuals aware of the transplantation law articles were relatively low ranging from 56% to 23%. Similar results of low awareness have been reported in other countries [25, 26]. It should be noted that the acceptance rate for the law articles was high (91% to 74%). Hence, improving the awareness of the current law should be considered by stakeholders. Nevertheless, individuals could agree with the articles without willing to donate their own organs.

Among the eleven consenting options presented to participants, one option involved not accepting organ donation. This option was the first choice for 22% of participants (highest mode). The ten other options were about different forms of consenting for those who agree to organ donation. Collectively, one of these ten options was the first choice for 78% of participants. So it is true that the option for refusing organ donation had the highest mode but at the same time those refusing organ donation accounted for only 22% of participants.

Regarding the most preferred consenting option, our results showed that the most favorable consenting system was the *Informed consent by donor only* while the least favorable was the *Presumed consent*. These findings could, at least in part, be attributed to the lack of trust in the health care system and the concern that medical personnel might be reluctant to provide utmost care to some patients. The reasons for refusing the presumed consent should be further investigated as this form of consent is –arguably- easier to implement and result in a higher willingness to donate [23]. The presumed consent system, however, has been the subject of major public and ethical debates because it may represent a violation of the right of autonomy, where the individual’s body would become a public property unless claimed otherwise [27]. Presumed consent could be considered by some as inaccurate and misleading and the actions based on it could not be reversed or undone [28]. Hence, presumed consent was not supported by the Institute of Medicine and was rejected by the American Medical Association’s Council on Ethical and Judicial Affairs, however, the British Medical Association produced a report supporting it [29]. Opposing evidence, however, was found in Belgium where there was an overall approval of presumed consent [30].

Our results also suggest that financial and medical incentives usually have a minimal effect on the individual’s decision to donate his/her organs. This suggests that participants perceived organ donation mainly as an act of altruism rather than an opportunity for financial benefit and that donation is an act of charity that should not be compensated by materialistic benefits. Consistent with our results, a study in Scotland found that only 21% agreed that a financial incentive should be used [31] and another study in Saudi Arabia found that only 0.6% of the respondents agreed to donate their organs after death for financial reasons [32].

The strengths of this study include its relatively large sample size, inclusion of participants representative of different socioeconomic and educational levels. On the other hand, limitations include the inability to generalize the data as it was obtained from two governorates and that the results were based only on those who were willing to participate.

## Conclusion:

From this study, it could be concluded that around half of the Egyptian population agree to posthumous organ donation. This percentage could be increased significantly by raising the awareness about how the process of donation could be regulated and how the patient’s right of decision could be protected. Participants specifically found the *Informed consent by the donor only* to be the most preferred form of consent for organ donation and that offering incentives have a limited role in the decision of organ donation.

## Data Availability

The datasets used and/or analysed during the current study are available from the corresponding author on reasonable request.
